# Supplementary material for: Selective Induction of Intrinsic Apoptosis in Retinoblastoma Cells by Novel Cationic Antimicrobial Dodecapeptides
Source: Pharmaceutics. 2022 Nov 18;14(11):2507. doi: 10.3390/pharmaceutics14112507 (PMC9694048; doi:10.3390/pharmaceutics14112507)
Supplement: Supplementary file 1 [file pharmaceutics-14-02507-s001.zip › pharmaceutics-1970162-Supplementary.pdf]

pharmaceutics

Supplementary Materials:

Selective Induction of Intrinsic Apoptosis in Retinoblastoma Cells by Novel Cationic Antimicrobial Dodecapeptides

Vishnu Suresh Babu, Atish Kizhakeyil , Gagan Dudeja, Shyam S. Chaurasia, Veluchami Amutha Barathi, Stephane Heymans, Navin Kumar Verma, Rajamani Lakshminarayanan and Arkasubhra Ghosh

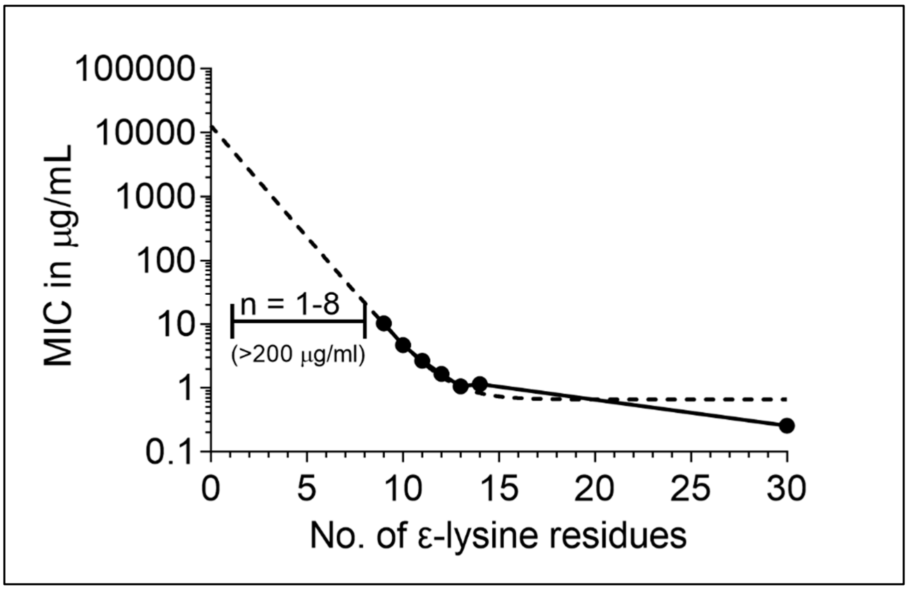

Figure S1. Correlation between MIC values and number of e-lysine residues. The dotted lines represent the non-linear fit and suggest the value is optimized at ≥12 residues.

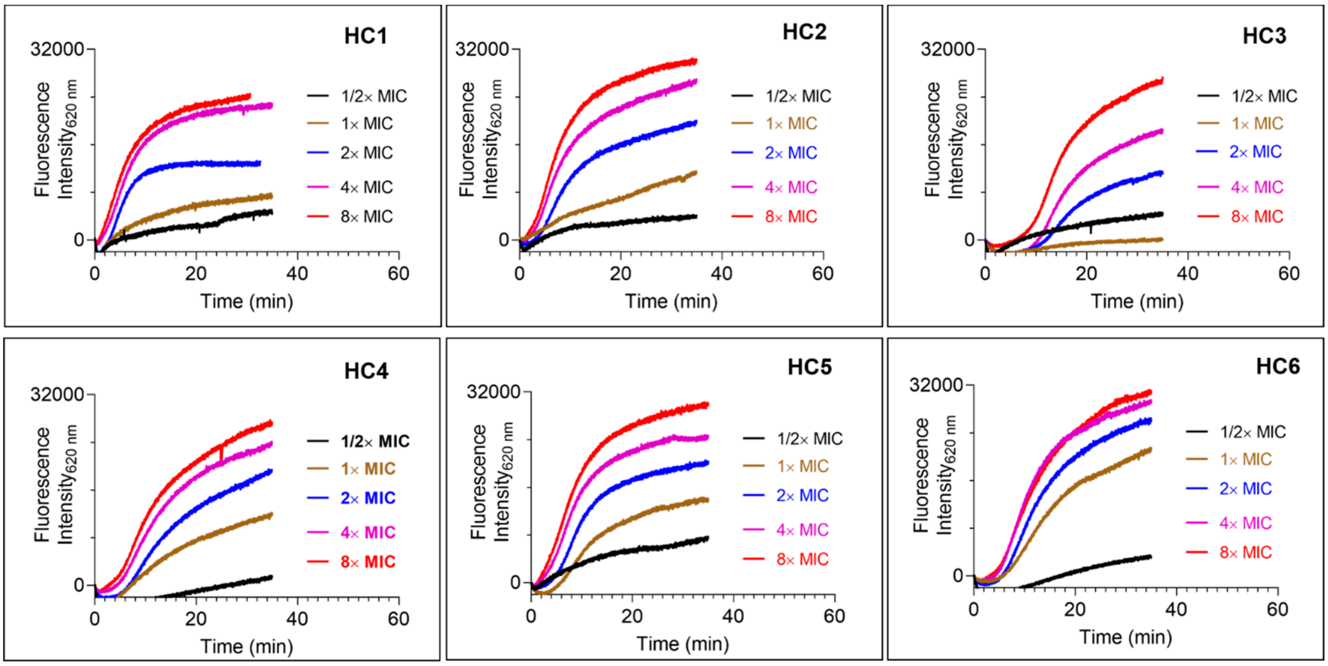

Figure S2. Interaction of peptides with the cytoplasmic membrane of *P. aeruginosa* ATCC 9027 strains probed by DiSC3-5 assay. Change in membrane potential of intact bacterial cells upon addition of peptides was inferred by the increase in fluorescence intensity of membrane-sensitivetive probe. The concentration of peptides was expressed in terms of MIC values.

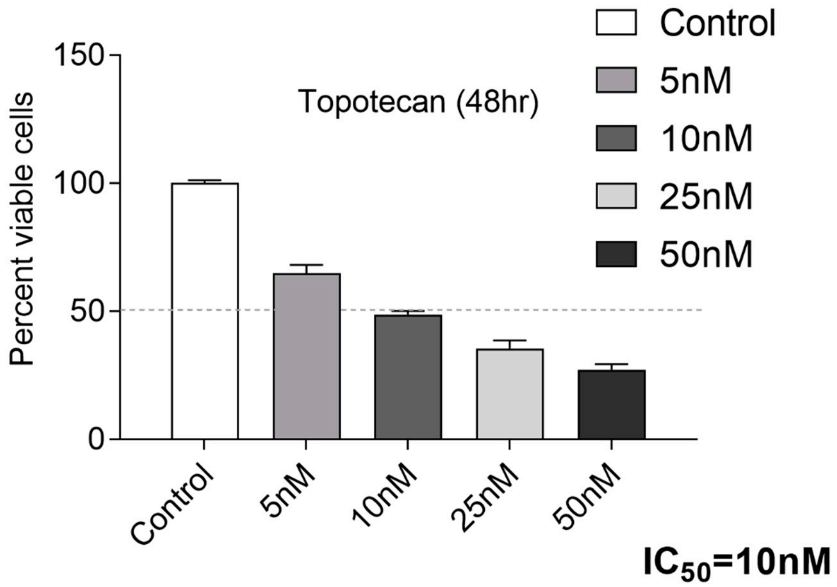

Figure S3. Determination of Topotecan IC_{50} in WERI-Rb1 cells using MTS assay. The dotted horizontal lines represent 50% viability. The concentrations of topotecan varied from 5nM to 50nM for 48hour exposure in WERI-Rb1 cells. Topotecan IC_{50} = 10nM for WERI-Rb1 cells.

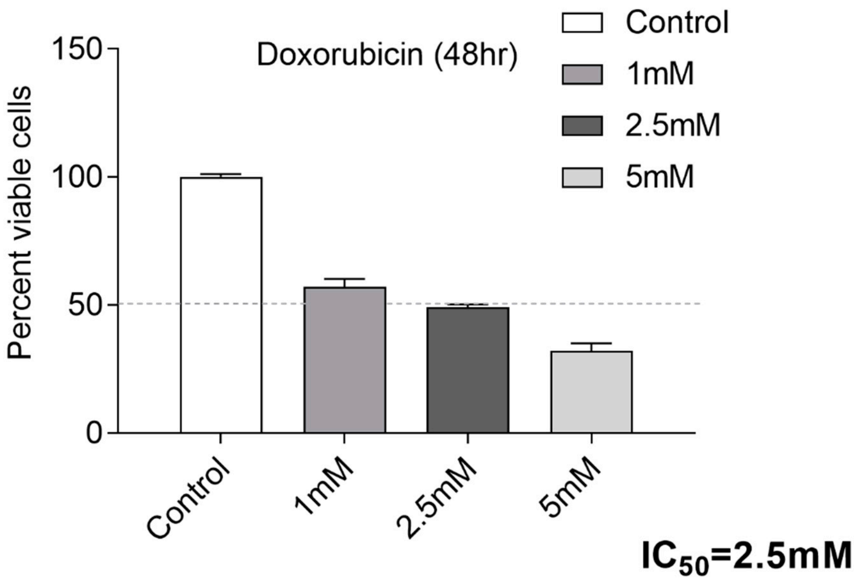

Figure S4. Determination of Doxorubicin IC_{50} in WERI-Rb1 cells using MTS assay. The dotted horizontal lines represent 50% viability. The concentrations of doxorubicin varied from 1mM to 5mM for 48hour exposure in WERI-Rb1 cells. Doxorubicin IC_{50} = 2.5mM for WERI-Rb1 cells.

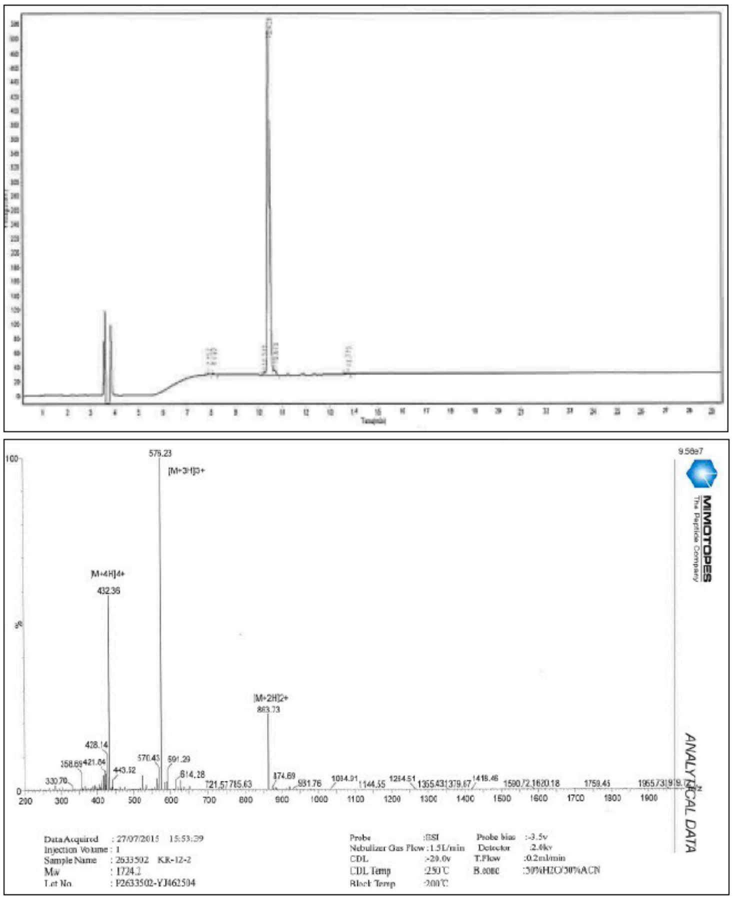

Figure S5. RP-HPLC (top panel) and ESI-MS (bottom panel) of peptide HC1.

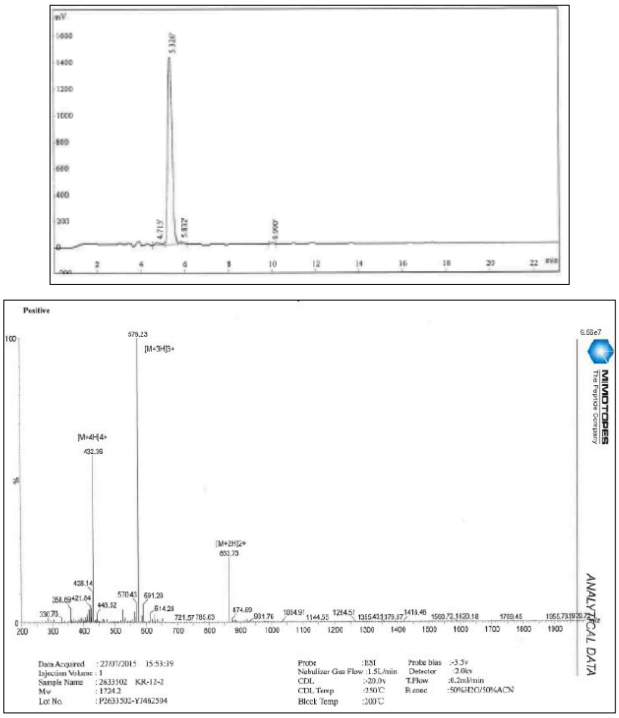

Figure S6. RP-HPLC (top panel) and ESI-MS (bottom panel) of peptide HC2.

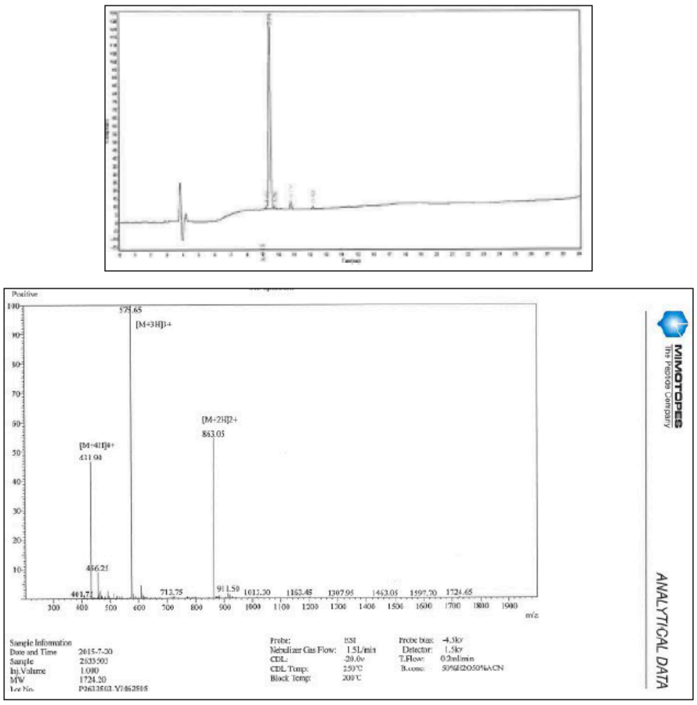

Figure S7. RP-HPLC (top panel) and ESI-MS (bottom panel) of peptide HC3.

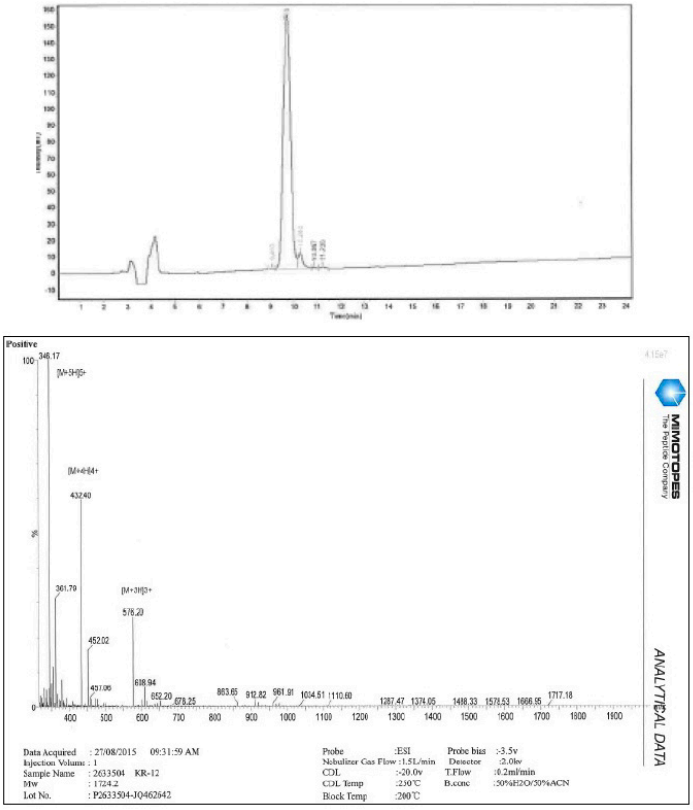

Figure S8. RP-HPLC (top panel) and ESI-MS (bottom panel) of peptide HC4.

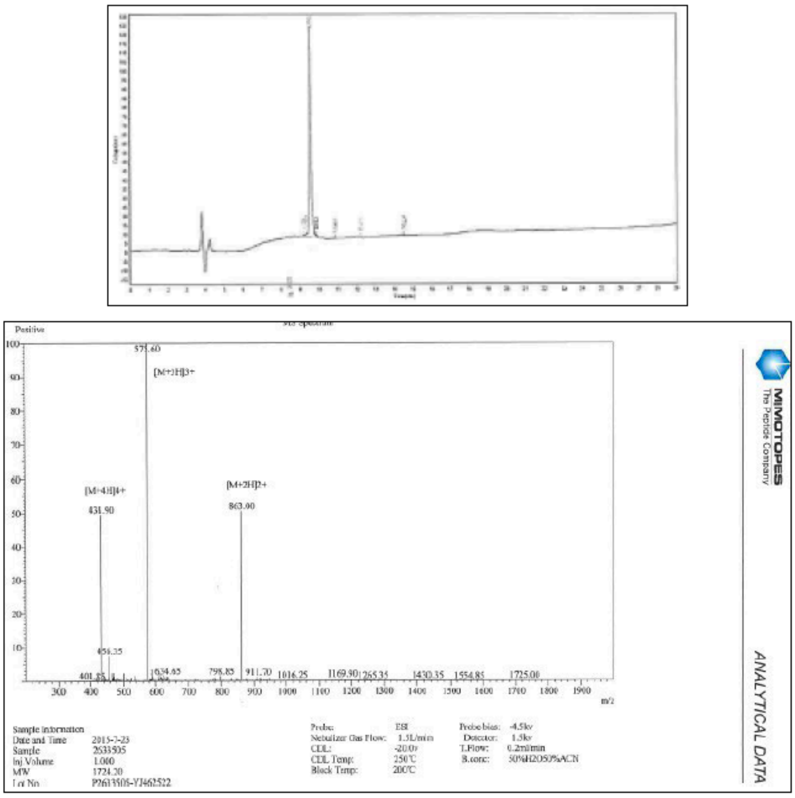

Figure S9. RP-HPLC (top panel) and ESI-MS (bottom panel) of peptide HC5.

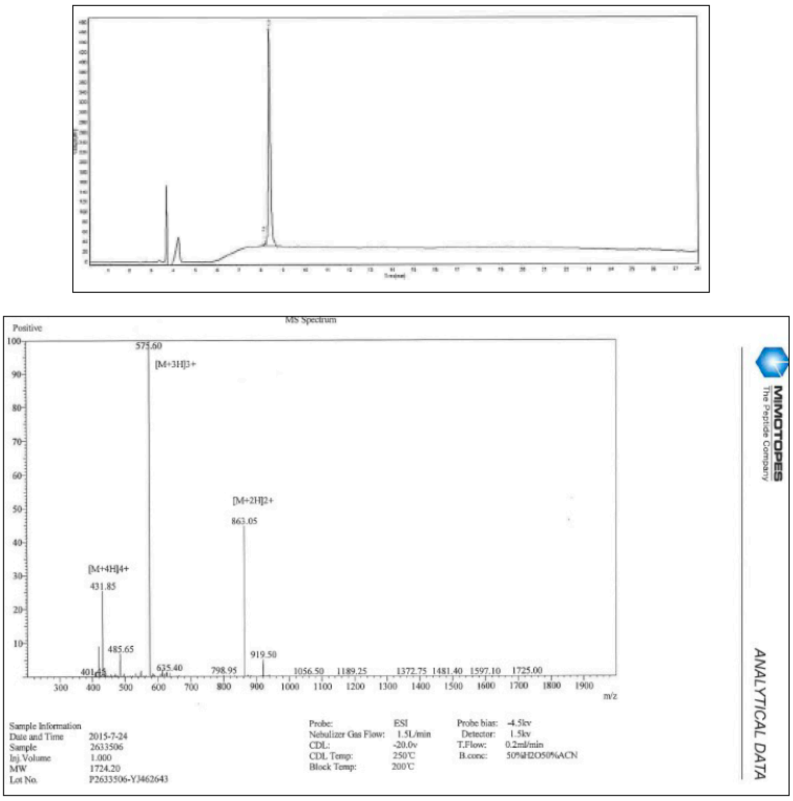

Figure S10. RP-HPLC (top panel) and ESI-MS (bottom panel) of peptide HC6.

Table S1. Antimicrobial properties of the peptides against a panel of P. aeruginosa and S. aureus/MRSA Strains.

Bacterial Strains	Minimum inhibitory concentration (μM) of peptides						
	**HC1**	**HC2**	**HC3**	**HC4**	**HC5**	**HC6**	**Melittin**
P. aeruginosa 27853	18.6	18.6	18.6	18.6	18.6	18.6	5.6
P. aeruginosa 15442	18.6	37.1	18.6	37.1	18.6	37.1	n.d.
P. aeruginosa 14476/07	18.6	37.1	18.6	37.1	18.6	37.1	n.d.
P. aeruginosa 23376	37.1	37.1	18.6	18.6	37.1	18.6	n.d.
P. aeruginosa 23155	74.2	37.1	18.6	18.6	18.6	37.1	5.6
P. aeruginosa 5790/07	37.1	37.1	18.6	18.6	18.6	37.1	n.d.
P. aeruginosa 4150R	37.1	37.1	18.6	18.6	18.6	37.1	n.d.
P. aeruginosa 01	9.3	74.2	9.3	18.6	9.3	37.1	5.6
P. aeruginosa 9027	9.3	9.3	9.3	18.6	9.3	18.6	2.8
P. aeruginosa 4877	18.6	18.6	18.6	18.6	9.3	18.6	n.d.
P. aeruginosa DM023257	18.6	37.1	37.1	18.6	18.6	18.6	n.d.
P. aeruginosa 01	37.1	18.6	18.6	18.6	18.6	18.6	5.6
P. aeruginosa 02	37.1	37.1	37.1	18.6	37.1	37.1	11.2
P. aeruginosa 03	37.1	37.1	37.1	18.6	18.6	18.6	5.6
P. aeruginosa 04	37.1	37.1	37.1	18.6	37.1	18.6	11.2
P. aeruginosa 05	18.6	18.6	18.6	18.6	18.6	18.6	5.6
P. aeruginosa 06	74.2	74.2	18.6	37.1	37.1	37.1	2.8
P. aeruginosa 07	18.6	18.6	18.6	18.6	18.6	18.6	2.8
P. aeruginosa 08	74.2	37.1	18.6	18.6	18.6	18.6	5.6
P. aeruginosa 09	18.6	37.1	18.6	18.6	18.6	18.6	2.8
P. aeruginosa 10	18.6	18.6	18.6	18.6	18.6	18.6	2.8
							**Daptomycin**
MRSA +B32:L426506	18.6	9.3	4.6	9.3	9.3	9.3	0.31
MRSA DB57964/04	9.3	9.3	9.3	9.3	9.3	9.3	0.31
MRSA 700699	37.1	18.6	18.6	9.3	18.6	9.3	0.62
MRSA BAA38	18.6	9.3	18.6	37.1	9.3	18.6	4.94
MRSA 43300	18.6	18.6	18.6	9.3	9.3	9.3	0.31
MRSA 42412	18.6	9.3	9.3	18.6	18.6	9.3	0.31
MRSA 68004	18.6	9.3	18.6	9.3	9.3	9.3	0.31
MRSA 9808R	37.1	18.6	18.6	18.6	18.6	18.6	0.31
MRSA 21595	37.1	18.6	18.6	18.6	18.6	9.3	0.31
S. aureus 4583R	37.1	37.1	37.1	18.6	18.6	37.1	0.31
S. aureus 6538	37.1	74.2	37.1	18.6	18.6	37.1	0.15
S. aureus 29213	18.6	37.1	37.1	18.6	18.6	18.6	0.15
S. aureus 29737	37.1	74.2	37.1	18.6	37.1	37.1	0.31
S. aureus 4400R	18.6	18.6	18.6	18.6	18.6	18.6	0.31
S. aureus 4299	18.6	18.6	18.6	18.6	18.6	18.6	2.47
